# The Beneficial Effect of Preoperative Exercise on Postoperative Clinical Outcome, Quality of Life and Return to Work after Microsurgical Resection of Spinal Meningiomas

**DOI:** 10.3390/jcm12082804

**Published:** 2023-04-10

**Authors:** Fatma Kilinc, Matthias Setzer, Vincent Prinz, Daniel Jussen, Gerhard Marquardt, Florian Gessler, Marcus Czabanka, Thomas Freiman, Daniel Dubinski, Sae-Yeon Won, Moritz Haberland, Bedjan Behmanesh

**Affiliations:** 1 Department of Neurosurgery, University Hospital Frankfurt, Schleusenweg 2-16, 60528 Frankfurt am Main, Germany; 2 Department of Neurosurgery, Goethe University Hospital, 60528 Frankfurt am Main, Germany; 3 Department of Neurosurgery, University Medicine Rostock, 18057 Rostock, Germany

**Keywords:** spinal menigioma, clinical outcome, return to work, quality of life

## Abstract

Objective: While outcomes of surgical treatment for spinal meningiomas are well-described within the literature, factors affecting early return to work as well as long-term health related quality of life remain unclear. Methods: In this retrospective study, patients with spinal meningioma and surgical treatment from two university-level neurosurgical institutions between 2008 and 2021 were analyzed. Time to return to work, physical activities and long-term health related quality of life (assessed by telephone interviews using the EQ-5D-5L health status measure and visual analogue scale (EQ VAS) were analyzed. Results: We identified a total of 196 patients who underwent microsurgical resection of spinal meningioma between January 2008 and December 2021. Of those, 130 patients of working age were included and analyzed. The median follow-up time was 96 months. All included patients returned to work. The median time of return to work was 45 days for the whole cohort. Patients who preoperatively performed physical activity returned to work significantly earlier compared to patients who did not (*p* < 0.001). Furthermore, younger age (*p* = 0.033) and absence of obesity (*p* = 0.023) correlated significantly with earlier return to work. Significant differences were also observed in all 5 EQ-5D-5L dimensions between patients with and without preoperative physical activity. Conclusions: Despite the benign nature of spinal meningioma preoperative physical activity and physiological body weight are associated with favorable postoperative outcome, higher quality of life and early return to work.

## 1. Introduction

Spinal meningiomas are slow growing benign tumors with a higher prevalence among women and which account for 25–30% of all spinal tumors [1,2]. Most meningiomas are located in the thoracic spine and present with local pain and myelopathy. Spinal meningioma can be safely resected without causing neurological damage and good functional outcomes can be achieved [3,4,5,6,7]. Following resection of a spinal meningioma, the ability to remain at or return to work (RtW) is in the best interests of the individual patient, society and employers. Early return to work may help to reduce financial losses for both employees and employers, decrease social isolation and improve self-esteem [8,9]. Health-related quality of life (HRQoL) and return to work in turn influence overall social satisfaction [10].

According to the World Health Organization (WHO), quality of life is defined as an individual’s perception of his/her position in life in the context of culture and according to the value systems of the society in which you live, and in relation to your goals, expectations, standards and concerns [11]. Life satisfaction is usually considered a separate aspect of quality of life, which also reflects functional limitation [12]. Whereas several studies have reported positive outcomes after surgery for spinal tumors, postoperative outcome was measured mainly by neurological deficits, total resection and progression-free survival while aspects of return to work and health-related quality of life remain largely unclear. The aim of this study is therefore to identify factors associated with good health-related quality of life and return to work following surgery for spinal meningiomas in order to determine possible risk factors for negative outcomes and in turn to increase treatment success in patients with spinal meningiomas.

## 2. Materials and Methods

**Figure 1:**
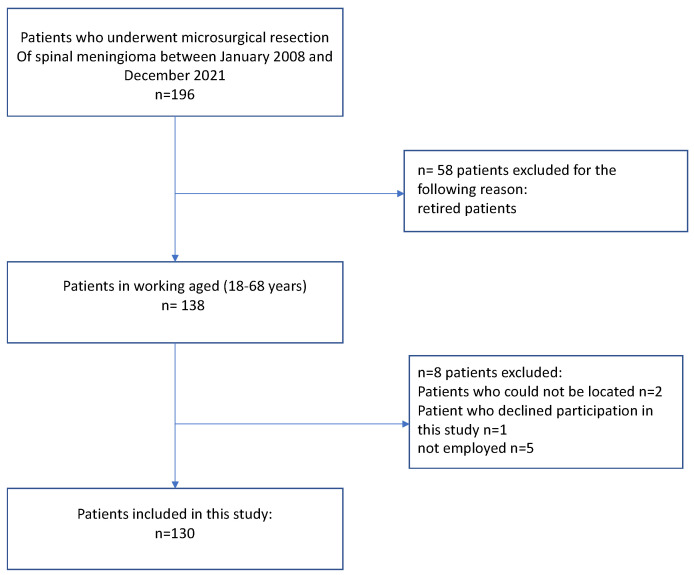
Flowchart describing the inclusion process of all patients who are actively employed at the time of surgery.

Patients undergoing resection for spinal meningioma at two university hospitals between 2008 and 2021 were retrospectively identified. In total, we analyzed 196 patients with spinal meningioma. Due to the aim of this study, patients who were retired at the time of surgery, missing follow-up, as well as patients who could not be located or refused to participate in this study and unemployed patients prior to spinal meningioma surgery were excluded. Overall, a total of 130 patients were included in the final analysis (Figure 1).

Patient characteristics, body mass index (BMI), Charlson comorbidity index (CCI), tumor location, number of affected spinal levels, initial symptoms, duration of symptoms until surgery, surgical approach, surgical time (time from skin incision to wound closure), surgeon’s experience (more and less than 10 years neurosurgical practice), recurrence rate and American Society of Anesthesiologists (ASA) Score, as well as tumor calcification were analyzed.

The neurological status at first presentation, postoperatively at discharge, as well as in long-term follow-up, were assessed using Eastern Cooperative Oncology Group (ECOG) Performance Score. The extent of resection (EOR) was classified according to the Simpson grading system. The EOR was assessed by the surgical report and additionally on early postoperative MRI during hospital stay. The degree of tumor resection was assessed as described previously [13].

Only to assess the factors determining an early return to work, standardized telephone interviews were conducted using a structured, one-time detailed questionnaire asking patients about their ability to work, time to return to work in days, employment after surgery, persistent symptoms, regular exercise before surgery and time to return to physical activity after surgery. In addition, preoperative occupation as well as employment status (full- or part-time work) were analyzed.

The patients were divided into three groups: those who exercised regularly before surgery, those who started exercising after surgery and those who did not exercise before or after surgery. Regular exercise was defined as practicing sports activities at least twice a week.

Health-related quality of life was assessed pre- and postoperatively during the last clinical follow-up with the EQ-5D-5L generic questionnaires and the EuroQol questionnaire visual analogue scale (EQ-VAS). Patients were asked to rate their current health state on a visual analogue scale between 0 and 100 with the endpoints “worst imaginable health state” to “best imaginable health state” [14]. In addition to the EQ-VAS the EQ-5 D description system includes five dimensions with one item per dimension: mobility (MO), self-care (SC), usual activities (UA), pain/discomfort (PD) and anxiety/depression (AD) [15].

This study was approved by the local ethics committee.

### Statistical Analysis

All statistics were performed using IBM SPSS (version 21, IBM Corp., Armonk, NY, USA); *p* < 0.05 was considered statistically significant. Comparison of differences between the study groups was made using Fisher’s exact test for categorical variables. Nonparametric tests include the Mann–Whitney-U-test and Kruskal–Wallis test to compare groups of data that did not follow the normal distribution. Tests for normality were performed using Shapiro–Wilk-test. Univariate analysis was performed to determine the effect of clinical variables on early return to work. In a second step, a multivariate analysis was performed to find independent determinants for early return to work by using a linear regression analysis.

## 3. Results

### 3.1. Patient Characteristics

Ultimately, 130 patients with a mean age of 51 years were included for final analysis. The sex distribution included 94 female (72.3%) and 36 male (27.7%) patients. The spinal meningiomas were most often located in the thoracic spine (60.7%) followed by cervical spine (31.5%). In most cases the tumor affected only one level (78%). Patient comorbidities were assessed according to the Charlson comorbidity index (CCI). In 41.5% the CCI was 0, followed by 1 (26.9%) and 2 (28.5%). In three cases (2.3%) the CCI-index was 3 and in one case (0.7%) 4. Tumor calcifications were observed in only 4 cases. The symptoms resulting in admission were sensory deficit (38.9%) and motor weakness (26.8%), followed by gait ataxia (22.7%) and back pain (15.9%). In 14 cases (16.9%) the tumor was discovered as an incidental finding, in 18 cases (13.8%) radicular pain was described and in eight cases (5.9%) bladder/bowl dysfunction led to hospital admission and consecutively to the diagnosis of the meningioma. Symptom duration until surgery was between 0–3 month in 36.9% of patients, followed by 4–6 months in 24.6% of patients. In 20 cases, patients had symptoms for more than 12 months. In a total of 16 patients (12.3%), the duration of symptoms was between 7 and 9 months. A total of 14 patients (10.8%) described no symptoms. Further details on patients with spinal meningiomas are given in Table 1.

### 3.2. Surgery

In 89 cases (64.8%), the meningioma was accessed by a laminectomy. In 38 cases (29.2%) a hemilaminectomy and in 3 cases (2.3%) laminoplasty was performed. Complete resection was achieved in 91.5% (Simpson grades I and II) and subtotal resection in 8.5% (Simpson III and IV). According to surgical reports, a dural attachment was observed to be dorsal in 33.8% of the cases, followed by dorsolateral (21.5%), lateral (20.8%), ventrolateral (13.8%) and ventral (10.0%). In most cases, the surgeon’s experience was greater than 10 years (94.6%). Histologically WHO grade 1 meningiomas could be detected in more than 90% of cases, Table 2.

### 3.3. Outcome

After a mean follow-up of 96 months, 124 patients (95.4%) had an ECOG performance status of 0. The remaining patients had an ECOG performance status of 1 (4.6%). None of the patients displayed an ECOG performance status of greater than 1. Thus, an improvement was observed in 113 patients (86.9%), stable neurological status was recorded in 17 patients (10.8%). Table 1 contains further patient data, Table 3.

### 3.4. Return to Work

After surgery, all patients returned to their previous occupations. In most cases patients performed office work (61.5%), followed by manual labor (20.0%) and intellectual work (18.5%). In assessing the pre- and post-operative occupations of our patients, we found a shift from part-time to full-time employment, Table 4. The majority (73.8%) returned to work within three months. Across the cohort, the median time to return to work was 45 days. Those patients who exercised regularly returned to work after a median of 30 days, while patients without regular exercise did not return to work until 130 days following surgery (*p* < 0.0001), Table 5.

After dichotomizing the variable for return to work, significant differences were found in the univariate analysis: weight (*p* = 0.033), age (*p* = 0.023), regular physical exercise before surgery (*p* < 0.001). The multivariate analysis showed that regular physical activity (RPE) before surgery and early resumption of physical activity after surgery were significant. The Simpson score, tumor calcification, ASA-Score, dural attachment, Charlson comorbidity Index, performed job, follow up ECOG and surgeon´s experience had no significant influence for early return to work, Table 6.

### 3.5. Sports

#### 3.5.1. Regular Physical Activity before Surgery

During the study, 90 (69.2%) patients reported regular physical activity before surgery. For this cohort in 62 cases (69%) patients performed individual sports and 28 patients (31%) participated in team sport. A total of 12 patients (19.4%) who performed individual sports practiced more than one individual sport activity. After surgery only 9% performed more than one individual sport and only 11% continued to practice team sports. Regarding the frequency of sports activities, most patients participated in sports activities two to four times per week before surgery, while 10.6% exercised more than five times per week. After surgery, intensive physical exercise more than 5 times a week decreased to 6% while other patients continued to be active 2–4 times/week.

A total of 64 patients (71%) who reported regular physical activity before surgery had a normal weight followed by 15 patients (16.7) with a BMI between 25.0–29.9 (overweight). In 8 cases (9%) a BMI between 30–34.9 (obese Class I) and in 3 cases (3.3%) patients were classified as obese Class II.

#### 3.5.2. Regular Physical Activity after Surgery

A total of 14 patients (35%) who do not perform exercise before surgery, started for the first time postoperatively. In this cohort patients performed individual sports, participation in team sports were not observed. Six patients performed more than one individual sport activity. Regarding the frequency of sports activities, all patients participated for one or two times per week (Table 7).

Five patients (71%) who started with regular exercise after surgery had a normal weight followed by 3 patients (16.7) who were overweighted (BMI between 25.0–29.9). Additionally, in 3 cases (9%), a BMI between 30–34.9 (obese Class I) and in 1 case (3.3%) patient were classified as obese Class II (Table 8).

Even in this cohort, compared to the no sport group, a significant difference was also found with regard to return to work (52 vs. 130 days, *p* = 0.001). A significant association was also found between occupation practiced and regular physical activity before surgery (*p* = 0.02) (Table 9). Office workers did more regular exercise before surgery than did manual laborers and intellectual workers (Table 10).

#### 3.5.3. Health Related Quality of Life (HRQoL)

The results of the EQ-5D-5L assessing health status overtime are summarized in Table 8 and shows that patients who do not exercise regularly have worse scores for mobility, self-care, usual activities, pain/discomfort and anxiety/stress than patients who exercise regularly (Table 11).

The results of the EQ-5D-5L assessing health status overtime are summarized in Table 12 and shows that patients who do not exercise regularly have worse scores for mobility, self-care, usual activities, pain/discomfort and anxiety/stress than patients who exercise regularly.

**Figure 2:**
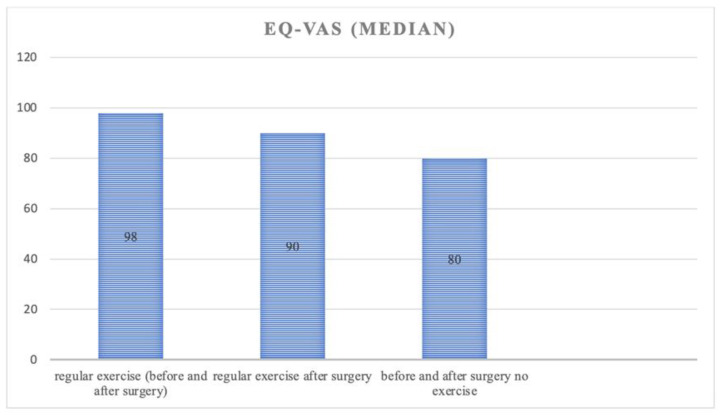
EQ VAS regard to exercise status.

Patients who exercised regularly before surgery had a higher general health score (EQ-VAS) compared to those who did not exercise before or after surgery. The cohort that started exercising after surgery had a lower score compared to the regularly active patients, but higher than patients who did not exercise before or after surgery (*p* = 0.001) Figure 2.

## 4. Discussion

Spinal meningiomas account for 25–30% of all spinal tumors and are usually benign and slow-growing tumors [1,2,3,4,5]. the postoperative outcome is generally described as good to very good [4,5,6,7]. However, as diagnosis and treatment have improved greatly over time, return to work especially early return to work and quality of life is an important outcome measure in spinal meningiomas. While there is sufficient data on resumption of occupation in patients with cranial meningiomas, and for a group of spinal ependymoma patients, this fact is little studied in patients with the spinal counterpart [16,17,18,19,20]. It has been shown that a large proportion of patients with cranial meningiomas do not return to work [20]. 

While the benefits of physical activity in patients with spinal cord injuries have been described and published, there are only few examples of the literature addressing the effect of physical activity and quality of life in spinal tumor patients are available [21,22].

The role of employment in our society has become a central feature of economic and social life. A survey from 2015 among the German population between the ages of 18 and 60 showed that work and being employed rank as an important factor in quality of life after family and partnership.

Therefore, it is particularly important to investigate this aspect in patients with spinal meningiomas.

A surprising effect in our study was that the timing of return to work was critically dependent on preoperative physical activity. This is a novel aspect and has received little attention to date. It has been shown that physical activities started preoperatively have a positive influence on professional reintegration. Rehabilitative effect of physical activity was already described for neuro-oncology patients, especially for brain tumor patients [23,24].

In addition to regular physical activity, our study shows significant factors such as age and weight had for earlier return to work.

As physical activity helps to regulate normal weight, the positive effects of maintaining a healthy body weight through physical activity are of particular importance with regard to postoperative recovery and reintegration into work. In our study this positive effect was also shown for overweight patients. A total of 90 patients performed regular physical activity before surgery. Overall, healthier patients who maintain a healthy weight and regular exercise are more likely to benefit from better postoperative outcomes, in our study nearly 30% were overweight. 

Even in the literature, regular physical activity with positive effects on metabolism and the cardiovascular system that persist even after a short interruption are described [25]. In addition, studies have shown a relationship between BMI-score and HRQL [26,27,28]. In 2013, Groeneveld et al. described a positive influence of physical activity on return to work and work performance in cancer survivors, as well as a positive influence of RtW on physical activity [29]. Hughes et al. also described that rehabilitation before major abdominal surgery involving respiratory and exercise interventions can reduce overall and pulmonary morbidity after surgery [30]. Therapeutic rehabilitation would certainly be supportive for patients who suffered from ataxia, weakness or paralysis prior to surgery.

A large percentage of patients continued to perform individual activities after surgery, but the number of those who did more than one sportive activity before surgery decreased. Additionally, the amount of participation in team sports decreased, which patients attributed in part to fear and subjective insecurity. Although the intensity of physical activity per week decreased after surgery a significant earlier return to work was observed. This was also observed for patients who started sports after surgery in comparison to those, who did no exercise either before or after surgery. However, it should be noted that regular physical activity is possible in patients with no or mild neurological impairment. A total of 69.2% of patients were regularly physically active before surgery. Most patients presented with sensory symptoms (38.9%) first, followed by motor deficits (26.8%). Nevertheless, the limited physical activity due to the neurological deficits may have been a reason for the early diagnosis. In most patients, symptoms duration until surgery was less than 3 months.

Regarding EQ-5D-5L, a significant difference was observed in all five dimensions for patients who exercise regularly in comparison to those who do not exercise. After resection of a spinal meningioma the ability to stay at work or return to work (RtW) is in the best interest of the individual, society and employers. An early return to work promotes a reduction in financial losses, reduces social isolation and improves self-esteem [8,9].

This is also evidenced by the results of the general health score (EQ-VAS), which indicated a higher self-described health status in the group that exercised regularly, both before and after surgery compared to those who never exercised or started exercising only after surgery. Health-related quality of life (HRQoL) and return to work affect overall social satisfaction. This could also be shown in all 5 dimensions of EQ-5D-5L. Here, a significant result was found for patients who practice regular sports before surgery.

In a previous study, we were able to show that obese patients experience more often postoperative complications, such as surgical site infections, prolonged length of surgery, venous thromboembolism or prolonged length of surgery, which affect the overall outcome. Weight reduction prior to planned surgery, when appropriate, should be recommended for patients with mild or absent symptoms attributable to spinal meningiomas to ensure not only a reduced rate of postoperative complications but also an early return to work and thus economic and social independence.

## 5. Conclusions

Microsurgical resection of spinal meningiomas is compared with a good outcome. Nevertheless, despite the benign nature of spinal meningioma this study shows that preoperative physical activity and normal weight influence positive outcome. Physical activity is also associated with higher quality of life and early return to work.

### 5.1. Limitations

This study is limited by its retrospective design. Most patients in our study had a long follow-up period. Our cohort might be subject to recall bias because of the time that elapsed between our evaluation and time of tumor resection. Possible approaches to improve participation in physical exercise were not analyzed. In addition, intensity and duration of different activities were not assessed. Furthermore, we only included patients that underwent surgery for analysis, thereby creating a selection bias. The ECOG performance status was used to describe symptom burden. In the case of benign disease with possible complex neurological impairment, an additional description using another index, e.g., the Karnofsky index, may be considered.

### 5.2. Disclosures

The authors report no conflict of interest concerning the materials or methods used in this study or the findings specified in this paper.

## Figures and Tables

**Table 1:** Patient characteristics.

	*n* (%)
Mean age, y (± SD)	51 ± 12.5
Male	36 (27.7)
Female	94 (72.3)
Tumor location	
Cervical	41 (31.5)
Cervicothoracic	5 (3.8)
Thoracic	79 (60.8)
Thoracolumbar	3 (2.3)
Lumbar	2 (1.5)
Affected level	
1	101 (78)
2	26 (20.4)
>2	3 (2.3)
Charlson Comorbidity Index (CCI)	
0	54 (41.5)
1	35 (26.9)
2	37 (28.5)
3	3 (2.3)
4	1 (0.7)
5	0 (0)
Symptoms and signs	
Sensory deficit	51 (38.9)
Motor weakness	35 (26.8)
Gait ataxia	30 (22.7)
Incidental finding	14 (16.9)
Back pain	21 (15.9)
Radicular pain	18 (13.8)
Bladder/bowl dysfunction	8 (5.9)
Symptoms duration until surgery	
0–3 months	48 (36.9)
4–6 months	32 (24.6)
7–9 months	16 (12.3)
10–12 months	14 (10.8)
>12 months	20 (15.4)
Calcification None	126 (96.9)
Partial	4 (3.1)
BMI	
18.5–24.9 (normal)	75 (57.7)
25.0–29.9 (overweight)	30 (23.1)
30.0–34.9 (obese Class I)	19 (14.6)
35.0–39.9 (obese Class II)	6 (4.6)

**Table 2:** Surgical data.

	*n* (%)
Surgical approach Laminectomy	89 (68.5)
Hemilaminectomy	38 (29.2)
Laminoplasty	3 (2.3)
EOR	
GTR	119 (91.5)
STR	11 (8.5)
Mean surgical time, min	183
ASA	
I	5 (3.8)
II	89 (68.5)
III	33 (25.4)
IV	3 (2.3)
Dural attachment	
Ventral	13 (10.0)
Ventrolateral	18 (13.8)
Lateral	27 (20.8)
Dorsolateral	28 (21.5)
Dorsal	44 (33.8)
Surgeon´s experience	
<10 years	7 (5.4)
>10 years	123 (94.6)
Histology WHO 1	
Meningothelial	101 (77.7)
Psammomatous	9 (6.9)
Fibromatose	6 (4.6)
Transitional	2 (1.5)
Angiomatous	1 (0.8)
WHO grade 2	11 (8.5)

**Table 3:** Outcome data.

	*n* (%)
Follow up (median, months)	96
ECOG grade on admission	
0	41 (31.5)
1	64 (49.2)
2	24 (18.5)
3	1 (0.7)
4	0 (0)
ECOG grade at the end of FU 0	124 (95.4)
1	6 (4.6)
2	0 (0)
3	0 (0)
4	0 (0)
Neurological status at the end of FU	
Stable	113 (86.9)
Improved	17 (13.1)

**Table 4:** Pre- and postoperative professional occupation.

	Preoperative Occupation	Postoperative Occupation	Change
Full time	108 (83.1)	112 (86.2)	+3%
Part time	22 (16.9)	18 (13.8)	−3%
Intellectual work	24 (18.5)	24 (18.5)	
Manual labor	26 (20.0)	26 (20.0)	
Office work	80 (61.5)	80 (61.5)	

**Table 5:** Time to return to work.

Time to Return to Work (RtW)	*n* (%)
<1 month	27 (20.8)
1–3 months	57 (43.8)
3–6 months	23 (17.7)
>9 months	23 (17.7)

**Table 6:** Univariate/multivariate analysis affecting early return to work after surgery.

	Univariate Analysis, *p* Value	Multivariate Analysis, *p* Value
Weight	0.033	0.627
Age	0.023	0.725
Simpson	0.698	0.887
Calcification	0.774	0.995
Return to exercise (months)	<0.001	0.003
Regular physical exercise	<0.001	0.036
ASA	0.109	0.734
Dural attachment	0.08	0.089
CCI	0.824	0.082
Performed Job	0.824	0.071
ECOG FU	0.129	0.139
Surgeon’s experience	0.098	0.099

RtE (Return to exercise) RPE (regular physical exercise).

**Table 7:** Type of physical activities.

Individual Activities	*n* (%)
Walking	10 (33.3)
Work outs/Fitness	15 (16.7)
Gymnastic	2 (20)
Bicycle riding	3 (12.2)

**Table 8:** BMI of patients who performed regulary exercise after surgery.

18.5–24.9 (normal)	5 (35.7)
25.0–29.9 (overweight)	3 (21.4)
30.0–34.9 (obese Class I)	4 (28.6)
35.0–39.9 (obese Class II)	2 (14.3)

**Table 9:** Type of physical activities before and after surgery.

	*n* (%)	
Individual activities	Before	After
Walking	30 (33.3)	21 (23.3)
Work outs/Fitness	15 (16.7)	14 (15.6)
Gymnastic	18 (20)	22 (22.4)
Bicycle riding	11 (12.2)	9 (10)
Team sport	15 (11.5)	
Golf/Tennis	2 (2.2)	1 (1.1)
Football	26 (28.9)	9 (10)

**Table 10:** Status of sports, RtS (return to sports) and RtW (return to work).

	*n* (%)
Sports/Excersice	
Regularly before surgery	90 (69.2%)
Regularly after surgery	104 (80.0%)
Time for RtS	
<1 month	83 (63.9)
1–3 months	15 (11.5)
3–6 months	5 (7.2)
6–9 months	1 (0.8)
>9 months	0 (0)
Return to work (median, days)	
Return to work, total	45
Return to work regular physical exercise	30
No physical exercise	130
Physical exercise after surgery	52

**Table 11:** BMI of patients who performed regular exercise.

18.5–24.9 (normal)	64 (71)
25.0–29.9 (overweight)	15 (16.7)
30.0–34.9 (obese Class I)	8 (9)
35.0–39.9 (obese Class II)	3 (3.3)

**Table 12:** Health-related quality of life EQ-5D-5L generic questionnaires related to regularly exercise vs. no sport before and after surgery.

EQ-5D-5L	Problem	Regular Exercise *n* = 90(%)	No Exercise *n* = 40(%)	*p* Value
Mobility	Level 1	88 (97.8)	29 (72.5)	<0.001
	Level 2	2 (2.2)	11 (27.5)	
	Level 3	0 (0)	0 (0)	
	Level 4	0 (0)	0 (0)	
	Level 5	0 (0)	0 (0)	
Self-care	Level 1	90 (100)	32 (80)	<0.001
	Level 2	0 (0)	5 (12.5)	
	Level 3	0 (0)	3 (7.5)	
	Level 4	0 (0)	0 (0)	
	Level 5	0 (0)	0 (0)	
Usual activity	Level 1	90 (100)	31 (77.5)	<0.001
	Level 2	0 (0)	5 (12.5)	
	Level 3	0 (0)	4 (10)	
	Level 4	0 (0)	0 (0)	
	Level 5	0 (0)	0 (0)	
Pain/discomfort	Level 1	90 (100)	30 (75)	0.001
	Level 2	0 (0)	8 (20)	
	Level 3	0 (0)	2 (5)	
	Level 4	0 (0)	0 (0)	
	Level 5	0 (0)	0 (0)	
Anxiety/stress	Level 1	86 (95.6)	30 (75)	0.001
	Level 2	4 (4.4)	9 (22.5)	
	Level 3	0 (0)	1 (2.5)	
	Level 4	0 (0)	0 (0)	
	Level 5	0 (0)	0 (0)	

Level 1—no problem; Level 2—slight problems; Level 3—moderate problems; Level 4—severe problems; Level 5—unable to do/extreme problems.

## Data Availability

The raw data supporting the conclusions of this article will be made available by the authors, without undue reservation.
